# Content of nutrients, trace elements, and ecotoxicity of sediment cores from Rożnów reservoir (Southern Poland)

**DOI:** 10.1007/s10653-019-00363-x

**Published:** 2019-07-06

**Authors:** Agnieszka Baran, Marek Tarnawski, Tomasz Koniarz, Magdalena Szara

**Affiliations:** 1 Department of Agricultural and Environmental Chemistry, University of Agriculture in Krakow, Al. Mickiewicza 21, 31-120 Kraków, Poland; 2 Department of Hydraulic Engineering and Geotechnics, University of Agriculture in Krakow, Al. Mickiewicza 24/28, 30-059 Kraków, Poland

**Keywords:** Sediment cores, Trace elements, Nutrients, Ecotoxicity

## Abstract

The aims of the study were to investigate the concentration of trace elements, nutrients, and ecotoxicity in bottom sediment cores collected from the silted part of the Rożnów reservoir (Southern Poland). Significant differences in the content of nutrients, trace elements, and ecotoxicity between five sediment cores were found. However, in the vertical distribution, there was no high variability of the above parameters, which means that the intensely suspended matter transported by the Dunajec river is and, at various times, has been homogeneous. Significant correlations between nutrients and trace elements (*r* = 0.33–0.91, at *p* ≤ 0.05) point to the same sources of the above-mentioned substances and similar levels of contamination in the sediment cores. However, the PCA results showed that cadmium and phosphorus in the sediment cores had different behaviors than other elements and can be associated mainly with anthropogenic sources. According to the degree of contamination factor, sediment cores fall under the category of considerable contamination of metals. Geochemical factors indicated that nickel, chromium, and cadmium (only sediment core *C*1) were found to be the cause of significant pollution in the sediment cores. Toxicity assessment found that most of the bottom sediment samples were classified as non-toxic or slightly toxic, only 10% of the sediment samples were toxic for *Vibrio fischeri*, and 6% of the samples were toxic for *Sinapis alba*. The two test organisms showed a different sensitivity, and higher toxic responses were recorded for *V. fischeri* than for *S. alba*. Cadmium and phosphorus were associated with toxicity for *S. alba* (*r* = 0.29–0.58, at *p* ≤ 0.05), whereas TOC, N, and S, and Ca for stimulation of growth this plants. Trace elements (*r* = 0.32–0.51, at *p* ≤ 0.05) and nutrients (S, K, Mg, Na, *r* = 0.44–0.58, at *p* ≤ 0.05) were positively correlated with inhibition of luminescence of *V. fischeri*. The studies of concentration and relation between trace elements, nutrients, and ecotoxicity are important in the ecological risk assessment and describing the quality of sediments with multiple sources contamination.

## Introduction

Vertical profile of chemicals in sediment cores has been recognized as a good indicator of the history of sedimentation of atmospheric and catchment pollutants (Kljakovič-Gašpič et al. 2008; Gao et al. 2018). Deposit cores may be an excellent tool for the assessment of anthropogenic and natural impact on water environments (Harikumar and Nasir 2010; Wang et al. 2012; Gao et al. 2018). Bottom sediments absorb all pollutants due to their structure, which makes them a natural geosorbent where pollutants introduced to the aquatic environment accumulate (Förstner and Salomons 2010; Ekere et al. 2017; Birch 2017). Moreover, their high ability to absorb and release dissolved substances causes the bottom sediments are key factor in nutrient recycling (Fafandel et al. 2015). Trace elements are specific pollutants that do not undergo biodegradation, which has a hazardous effect on the ecological balance in aquatic ecosystems. On the other hand, prolonged periods of excess nutrients may lead to eutrophication (Jung 2017). Eutrophication processes can cause several adverse effects, such as mass development and blooms of algae, production of cyanotoxic toxins, accumulation of submerged and floating organic material in the water, summer oxygen depletion, and lowering pH in the overflow water (Wilk-Woźniak and Mazurkiewicz-Boroń 2003; Jung 2017). In the aquatic environment, there is evidence that eutrophication could have an influence on content of trace elements in the bottom sediments. Changes in pH and redox conditions in the water–sediment system lead to the release of metals bound to exchangeable carbonate and easily reducible phases in the sediment (Helios-Rybicka and Wilson 2000; Kumar et al. 2015; Wang et al. 2017). Moreover, the high content of organic matter and nitrogen are considered as important agents for the assessment of the eutrophication rate. Cycle of organic matter (sediment organism consumption and mineralization) provides a long-term source of contamination affecting the food chain (Fafandel et al. 2015). Furthermore, various studies have reported that eutrophication and pollution by heavy metals are two key aquatic environmental problems (Wenchuan et al. 2001; Skordas et al. 2015).

Most studies on the environmental quality of bottom sediments were focused on that high content of trace metals can be responsible for sediment ecotoxicity and have impact on benthic organisms (Baran and Tarnawski 2015; Ilkova et al. 2018). However, at low content of pollution, other factors such as organic matter, nutrients, and ammonia concentration may be responsible for low to moderate toxicity (Czerniawska-Kusza et al. 2006; Łukawska-Matuszewska et al. 2009; Janke et al. 2011; Fafandel et al. 2015). Han et al. (2016) found that both nutrients (P) and trace elements (As) released from bottom sediments to the overlying water can have toxic impacts on aquatic ecosystems. In turn, Gong et al. (2001) and Baran et al. (2016) observed that higher level of nutrients in bottom sediment might hide the inhibitory impact of contaminants on sediments and hence give a false-negative assessment. Therefore, determination of the ecotoxicity of sediments based only on chemical parameters is difficult. Biotests can indicate the biological responses to the different chemical stresses (Baran and Tarnawski 2015; Gao et al. 2018). Furthermore, the advantages of bioassays over chemical quality assessment are that biological testing integrates the effects of all chemical substances present at their actual bioavailability and detects possible combination, synergistic, or antagonistic effects (Tarnawski and Baran 2018). Therefore, for a comprehensive evaluation of the quality of bottom sediments, pollution content, nutrients status, and ecotoxicological analyses should be an integrated.

The main aims of the study were: (1) to determine the distribution of nutrients, trace elements, and ecotoxicity along the five cores collected from the silted part of the Rożnów reservoir (Southern Poland); (2) to find the degree of contamination with trace elements; (3) to correlate the ecotoxicity distribution with nutrients and trace elements content in vertical profiles of the sediments.

## Materials and methods

### Study area

**Fig. 1:**
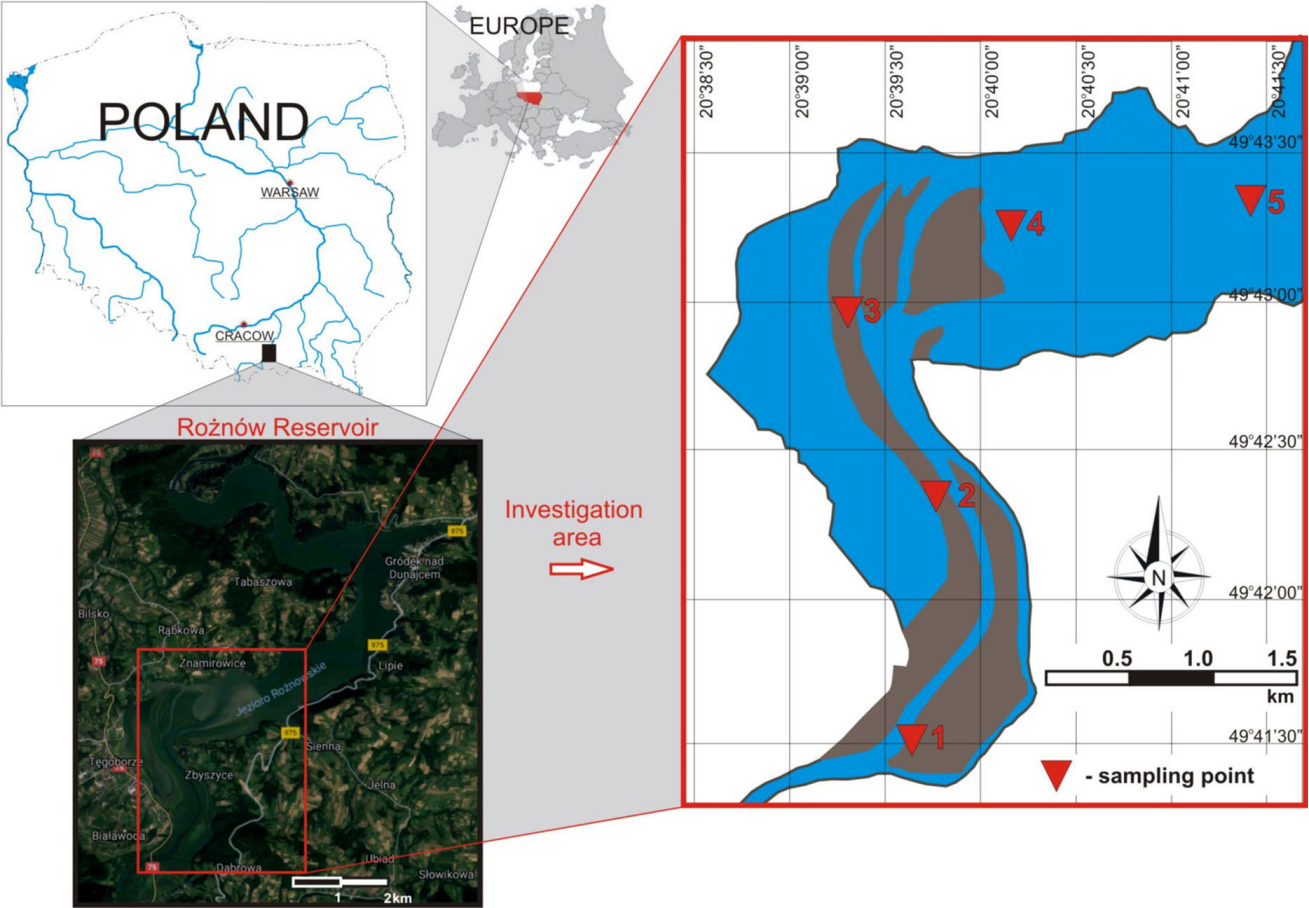
Location of the Rożnów reservoir and sampling sites

The Dunajec River, on which the Rożnów reservoir is located, is the second largest tributary of the Vistula River, draining the highest mountain massifs in southern Poland, as well as their parts beyond its borders (Fig. 1). The total area of the river basin is 6798 km^2^, and the length of the river is 250.3 km. The river flows range from 2.2 m^3^/s to the values of flood flows of 1700 m^3^/s (1934) and 1383 m^3^/s (1997) (Łagosz 2003). The basic tasks of the Rożnów reservoir are reducing the culmination of flood waves, production of electricity, improving the conditions of navigation, and providing municipal and industrial water. The reservoir was built in the period 1935–1941, interrupted by the outbreak of World War II (Table 1). In geological terms, the upper part of the river basin is made of a thin layer of alluvium on bedrock, and the remaining part is made of gravels and silty sediments. Such a structure determines the high intensity of surface and channel erosion processes causing silting of water reservoirs. From the very beginning of the reservoir use, an intensive process of capacity loss has been noticed, especially in the area of the estuary of the Dunajec River to the reservoir. The average annual value of the mineral material retained varies from 470 thousand m^3^/year to 2150 thousand m^3^/year and is the largest in Poland. Transporting the material by the river for many years resulted in the formation of outwash, islands, and the creation of a new river bed separating the side bay (Fig. 1). The renovation works undertaken have a local effect and do not solve the problem of the reservoir degradation comprehensively.

**Table 1:** Characteristic features of dam reservoir

Parameter	Values	Parameter	Values
Catchment area	4865 km^2^	Water exchange	11.1 times/year
Dam type/height	Concrete/49 m	Initial capacity	228 mln m^3^
Area of the reservoir	16 km^2^	The present capacity	~ 155.7 mln m^3^
Length	22 km	Sediments index	0.267 mm/year
Deep	0.5–30 m	Average annual loss of capacity	0.47%
Trophy	Eutrophic, rheolimnetic	Average annual sediment deposited	1298 thousands m^3^/year

### Sediment sampling

The Dunajec estuary to the Rożnów reservoir is an area of large deposition of bottom material. The research is limited to the most transformed part of the reservoir. Five core samples (Fig. 1) were collected there. The works were carried out from a floating platform using Instorf and Beeker samplers, and borehole piping was used. The cores were taken to the depth of 5 m below the reservoir bottom. The extracted bottom sediment cores with intact structure were divided into 0.5-m-long sections. Then they were packed, transported, and stored at 4 °C until conducting analyses. In the laboratory, the samples were air-dried at room temperature, then ground with a mortar and a pestle, and sieved through a 2-mm sieve.

### Chemical analyses

In core samples of the sediments, pH in 1 mol KCl dm^−3^ using the potentiometric method was determined. The content of carbon, nitrogen, and sulfur was determined using the Vario Max Cube analyzer. Total element (Zn, Cd, Pb, Cu, Ni, Cr, Fe, Mn, P, Ca, K, Mg, and Na) content in the sediments was determined using the inductively coupled plasma optical emission spectrophotometer (ICP-OES) Optima 7300 DV model by Perkin-Elmer after hot digestion in a mixture of HNO_3_ and HClO_3_ (3:2 v/v) acids (suprapure, MERCK) (Baran et al. 2016). During the chemical analyses, each sample of the bottom sediment was analyzed twice. The analytical results of the quality control samples showed a good agreement with the certified value reference material Trace Metals—Fresh Water Sediment CRM 16-050G and Soil Standard Loamy (OAS) batch no. 133505 (CNS), recoveries ranging from 71 (Cd) to 102% (Cr) and 90 (N) to 105% (C).

### Sediments contamination index

The classification of pollution of the sediments with trace elements was based on Bojakowska’s geochemical quality classes of bottom sediments (2001). The sediment quality guidelines (SQGs) of the numerical indices: threshold effect concentration (TEC) and probable effect concentration (PEC), were also used to assess potential hazard to organisms connected with the content of trace elements in the sediments using Macdonald et al. (2000). TEC values (Zn—121 mg; Cu—31.6 mg; Pb—35.8 mg; Cd—0.99 mg; Ni—22.7; Cr—43.3 mg kg^−1^) are used for the identification of contamination below which harmful effect on benthic organisms is not expected. PEC values (Zn—459 mg; Cu—149 mg; Pb—128 mg; Cd—4.98 mg; Ni—48.6; Cr—111 mg kg^−1^) are intended for identification of concentrations which, when exceeded, can have negative impacts on benthic organisms (Macdonald et al. 2000). Moreover, selected single and complex sediments contamination indices: anthropogenic factor (AF), contamination factor (CF), and contamination degree (CD) of trace elements in the cores, were calculated (Obhodas et al. 2006; Kljakovič-Gašpič et al. 2008; Natesan and Seshan 2010; Baran et al. 2016; Al-Mur et al. 2017). The CF was estimated as a ratio between the content of a particular metal in sediment and its geochemical background (Zn = 48 mg, Cu = 6 mg, Ni, Cr = 5 mg, Pb = 10 mg, Cd = 0.5 mg kg^−1^ d.m.) (Bojakowska 2001). The four categories were used to assess the level of trace elements contamination: CF < 1 low contamination; 1 ≤ CF < 3 moderate contamination; 3 ≤ CF < 6 high contamination; CF ≥ 6 very high contamination. The contamination degree (CD) was assessed based on the sum of all contamination factors and four categories were used to assess the total degree of contamination: CD < 8 low contamination; 8 ≤ CD < 16 moderate contamination; 16 ≤ CD < 32 considerable contamination, and CD ≥ 32 very high contamination (Tavakoly Sany et al. 2012). The AF was calculated as the ratio of trace element content in the surface sediments (0–0.5 m) to the content of trace elements at the depth in the sediment column (4.5–5 m). The value of AF > 1 for a trace element indicates that contamination exists, while if AF value is ≤ 1, there is no trace element enrichment of anthropogenic origin (Natesan and Seshan 2010).

### Ecotoxicity tests

Bottom sediment toxicity was determined using the Phytotoxkit and Microtox bioassays (Baran and Tarnawski 2013; Gao et al. 2018; Fafandel et al. 2015). *Sinapis alba* was used in Phytotoxkit, and the measurement parameters were inhibition of seed germination (IG) and root length inhibition (IR). The test was conducted in accordance with the procedure recommended by the manufacturer (Phytotoxkit 2004). The percent inhibition of seed germination (IG) or inhibition of root growth (RGI) for the *S. alba* was calculated with the formula:

$${\text{IG}}\;{\text{or}}\;{\text{RGI }} = \, \left[ {\left( {a - b} \right)/a} \right] \, 100\% ,$$

where *a* is the mean seed germination or root length in the control, and *b* is the mean seed germination or root length in the test sediment.

Microtox utilized the bioluminescent properties of *Vibrio fischeri*. In this test, a decrease in luminescence of the bacterium in an experimental sample is compared to that of the control. A standard test procedure—81.9% screening test, was applied (Baran et al. 2016). Luminescence was measured before and after a 15-min incubation of the bacterial suspension with the studied sample. The analysis of the change in luminescence was performed on a Microtox M500 Analyzer (Microbics Corporation 1992). The percent inhibition of luminescence (LI%) of *V. fischeri* was calculated with the formula:

$${\text{LI }} = \, \left( {1 - {\text{ls}}/{\text{lc}}} \right) \, 100\% ,$$

where ls is the intensity of luminescence of the studied sample; lc is the intensity of luminescence of the reference sample.

The tests were replicated three times. Toxicity results were expressed as percent effect (PE) and evaluated by following toxicity criteria: non-toxic samples percentage effect (PE) < 20%; slightly toxic samples 20% ≤ PE < 50%; toxic samples 50% ≤ PE < 100%; highly toxic samples PE = 100% (Persoone et al. 2003). Moreover, germination index (GI) for the *S. alba* was calculated according to the equation:

$${\text{GI }} = \, \left( {{\text{gs}} \cdot {\text{rs}}} \right)/\left( {{\text{gc}} \cdot {\text{rc}}} \right)100\% ,$$

where gs and rs are seed germination (%) and root elongation (mm) for the sample, respectively, and gc and rc are the corresponding control values. The results were classified according to the following criteria: 90–110% “no effect/non-toxic”, GI values < 90% inhibition, and GI values > 110% stimulation (Czerniawska-Kusza et al. 2006).

### Statistical analysis

All results were reported as the mean ± standard deviation and coefficient of variation (CV%). One-way ANOVA was used to assess the significant differences in analyzed parameters among five sediment cores. Spearmen’s correlation coefficient and principal component analysis were used to determine relationship and behavior of trace elements, nutrients, and ecotoxicological parameters in the bottom sediment cores. Statistica version 12.0 software and Microsoft Excel package were used to carry out the above analysis.

## Results and discussion

### Basic chemical properties and nutrient contents

**Fig. 2:**
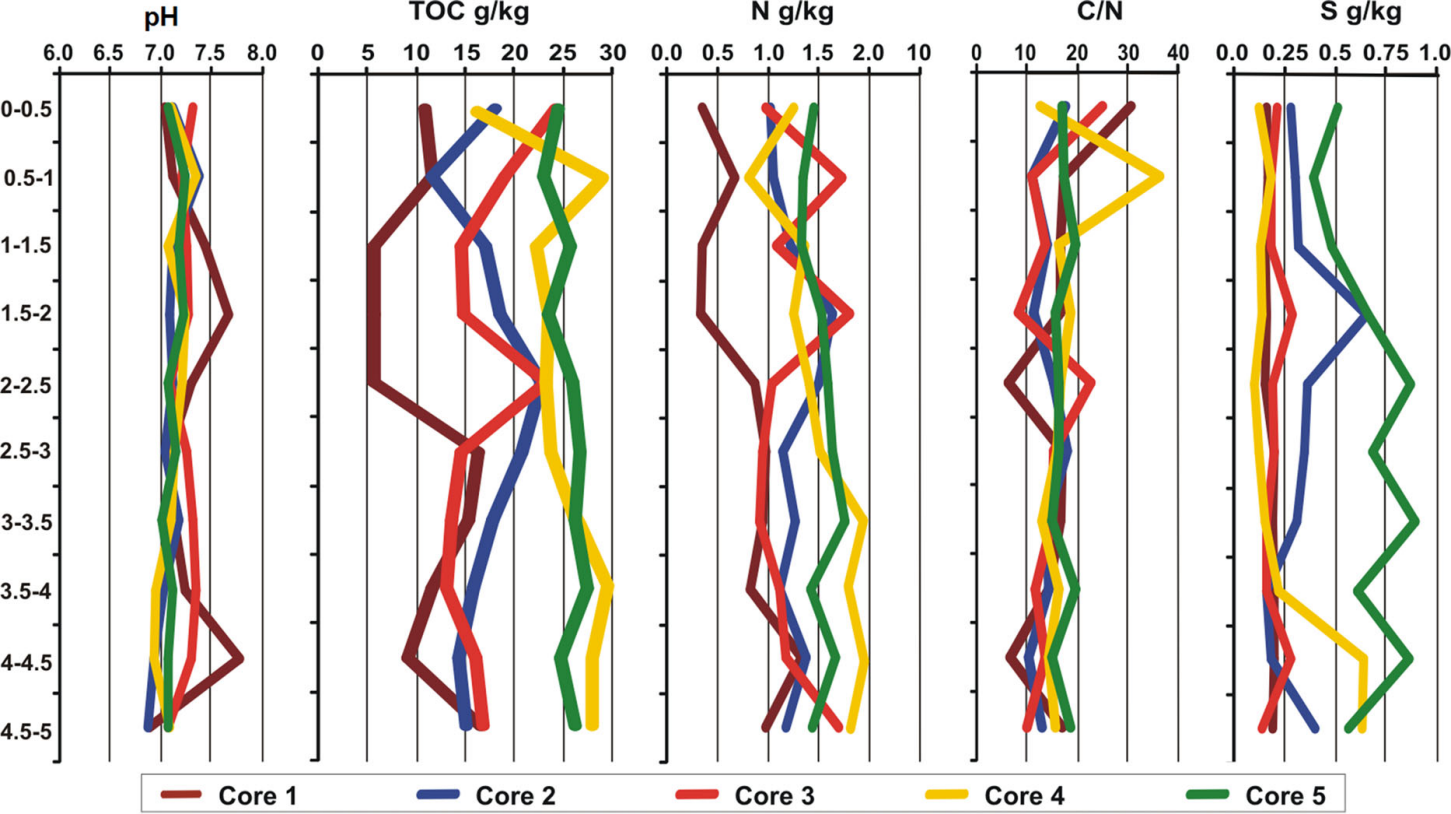
Vertical profile of content of TOC, N, S, and *C*/*N* ratio in the sediment cores

The obtained results point to significant variations in the parameters (C, N, S, *C*/*N*) of sediments collected from five deposit cores, except pH (Table 2, Fig. 2). The pH exhibited a very low degree of variability, indicated by the value of coefficient of variation between 1 and 3%. The pH for all subsamples in the core was between slightly acid and slightly basic. The content of TOC in the core samples varied from 5.47 to 29.5 g kg^−1^ d.m. A significantly higher content of TOC was found in cores *C*5 and *C*4, the lowest carbon content was observed in core *C*1. However, the greatest variability in TOC content was observed in vertical *C*1 (CV = 66%) and *C*3 (CV = 60%) the lowest—in vertical *C*5 (CV = 11%). Depending on depth, the highest content of TOC was recorded in bottom sediments in core *C*4 at a depth 0.5–1 m and 3.5–4 m. The mean content of N and S in the cores ranged between 0.33 and 2.01 g kg^−1^ d.m., and between 0.10 and 0.89 g kg^−1^ d.m., respectively. Significantly the highest content of both nutrients was observed in core *C*5: *C*5 > *C*4 > *C*2 > *C*3 > *C*1 (N) and *C*5 > *C*2 > *C*4 > *C*3 > *C*1 (S). Moreover, it was observed that vertical distribution of nitrogen corresponded with distribution of TOC in all cores. In the vertical profile, the highest content of nitrogen was observed in core *C*4 at a depth of 3–3.5 m and sulfur—in core *C*5 at a depth of 2–2.5 m and 3–3.5 m. Moreover, vertical distribution of S content was more varied than N content in the sediment cores (Fig. 2). The computed coefficients of variation (CV%) were for sulfur 24 (*C*1)–89% (*C*4) and for nitrogen 15 (*C*5)–64% (*C*4), respectively. Organic carbon-to-nitrogen ratios (*C*/*N*) in the study were between 6 and 36 (Fig. 2). The highest mean value of *C*/*N* ratio was found in cores *C*4 and *C*5 (17), and the lowest in cores *C*2 and *C*3 (14).What is more, we observed that surface subsamples in all cores had higher *C*/*N* variability compared to the bottom subsamples. High *C*/*N* ratios (15 or higher) in sediments indicate contribution of terrigenous organic carbon. Phytoplankton and zooplankton are rich in nitrogen compounds, and low *C*/*N* ratios (5–9) of sediments indicate a dominance of autochthonous organic matter (Burone et al. 2003). In the studied sediments, terrigenous organic matter dominates (Fig. 2). We observed that the *C*/*N* ratio was lower than 9 only in core 1 at a depth of 2–2.5 m and 4–4.5 m.

**Table 2:** Basic properties and nutrients content (g kg^−1^ dm) in the core sediments

Parameter	*C*1	*C*2	*C*3	*C*4	*C*5
pH	7.2 ± 0.40a	7.11 ± 0.23a	7.24 ± 0.29a	7.15 ± 0.15a	7.13 ± 0.09a
TOC	11.1 ± 7.55a	17.4 ± 7.47a	17.1 ± 9.89a	24.5 ± 5.84b	25.4 ± 2.74b
N	0.81 ± 0.51a	1.25 ± 0.50b	1.24 ± 0.79b	1.44 ± 0.45b	1.51 ± 0.22b
S	0.18 ± 0.04a	0.34 ± 0.25a	0.20 ± 0.14a	0.21 ± 0.19a	0.64 ± 0.28b
P	0.91 ± 0.41c	0.63 ± 0.25ab	0.62 ± 0.25ab	0.51 ± 0.10a	0.56 ± 0.06a
Ca	14.7 ± 6.50a	18.9 ± 8.09b	14.8 ± 7.05a	17.7 ± 2.40ab	21.4 ± 2.36b
Mg	4.92 ± 2.06a	6.35 ± 1.83bc	4.84 ± 1.51a	5.58 ± 0.98ab	7.01 ± 0.62c
K	2.39 ± 1.60a	3.94 ± 1.61b	2.56 ± 1.34a	2.79 ± 0.93a	4.01 ± 0.90b
Na	0.19 ± 0.07ab	0.24 ± 0.06b	0.16 ± 0.07a	0.15 ± 0.04a	0.21 ± 0.04b

Date expression: mean ± standard deviation, a, b, c indicted significant differences between cores (*p* < 0.05)

**Fig. 3:**
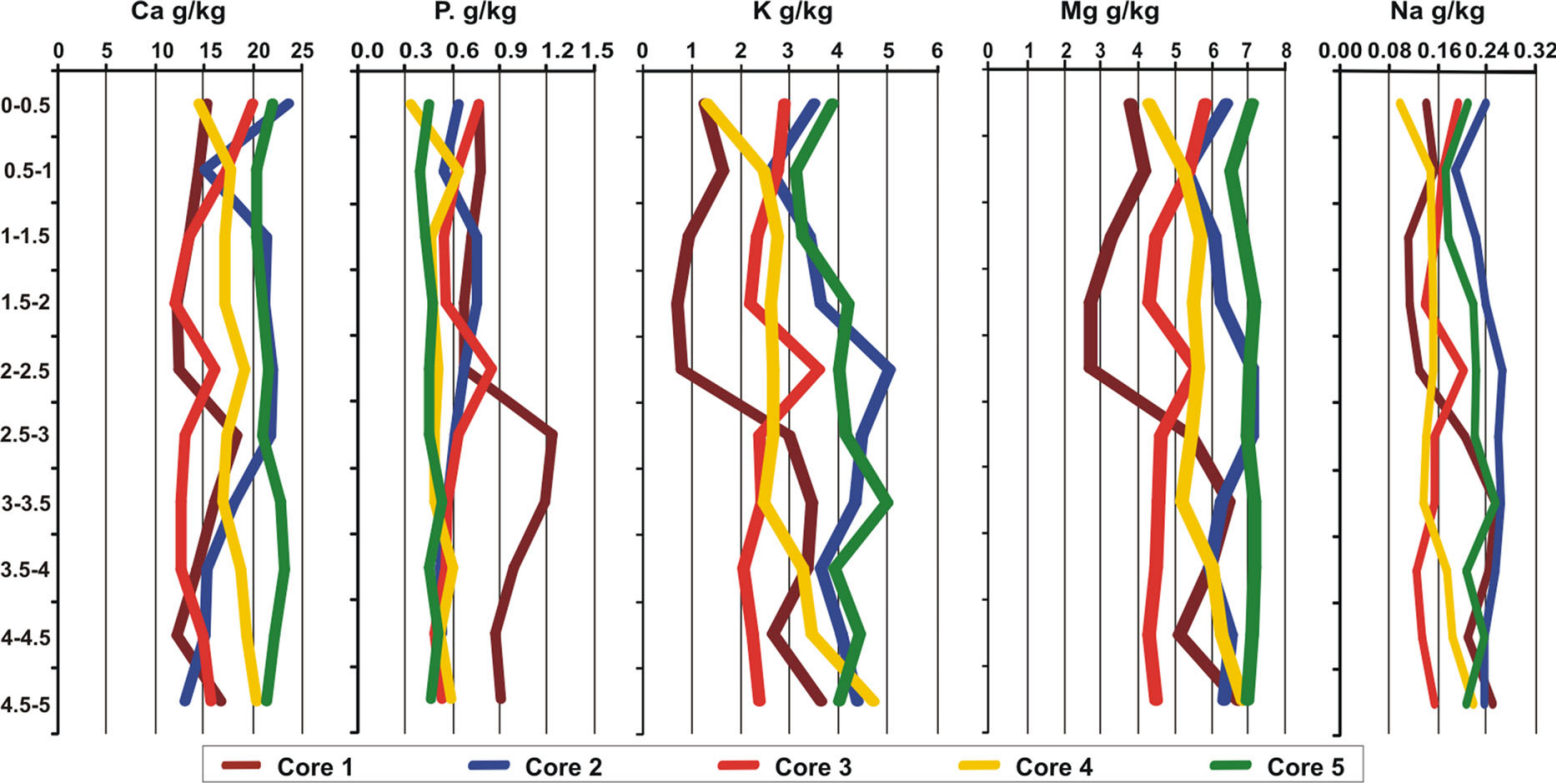
Vertical profile of concentration of Ca, P, K, Mg, Na in the sediment cores

Macronutrient concentrations (Ca, P, K, Mg, and Na) in the sediment cores are shown in Table 2 and Fig. 3. Significantly the highest content of Ca, Mg, K, and Na was found in core *C*5 and *C*2, and for P—in core *C*1. The highest concentration of nutrients was found at depths of 4.5–5 m and 5–5.5 m (K, Mg); 0–0.5 m and 5–5.5 m (Ca); 2.5–3 m and 3–3.5 m (P), and 3–3.5 and 4.5–5 (Na) (Fig. 3). However, the vertical profile of nutrients (K, Mg, P, and Na) in the sediment cores was similar (Fig. 3). The highest variability in the content of nutrients were found in cores *C*1 (CV = 38 Na—67% K) and *C*3 (CV = 31 Mg—52% K), and the lowest—in core *C*5 (CV = 9 Mg—22% K). The value of coefficients of variation (CV%) for nutrition were in the following order: K > Ca ≈ P > Na > Mg. The relatively low values of CV% suggest a small vertical variation in the content of these parameters.

### Assessment of concentration trace elements in the sediment cores

**Fig. 4:**
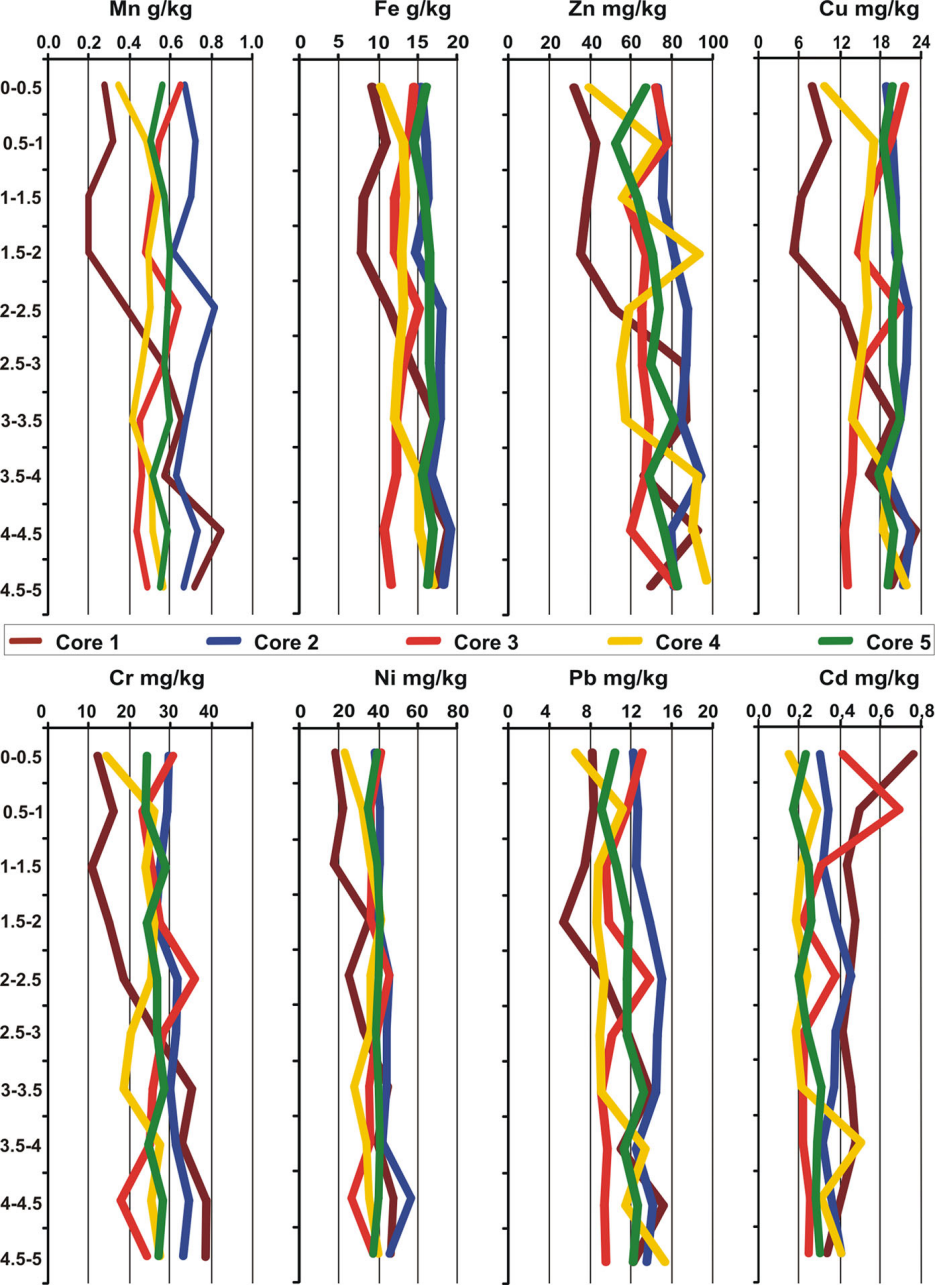
Vertical profile of trace elements concentration in the sediment cores

Concentration of trace elements in the sediment cores is presented in Table 3 and Fig. 4. The statistically significant highest content of metals was found in core *C*2, and Cd—in core *C*1, taking the following order: *C*2 > *C*3 > *C*4 ≈ *C*5 > *C*1 (Cr); *C*2 > *C*5 > *C*3 > *C*4 > *C*1 (Ni, Mn), *C*2 > *C*5 > *C*3 ≈ *C*4 > *C*1 (Cu); *C*2 > *C*3 ≈ *C*4 > *C*5 > *C*1 (Zn); *C*1 > *C*2 > *C*3 > *C*4 > *C*5 (Cd); *C*2 > *C*5 > *C*3 > *C*1 ≈ *C*4 (Pb); *C*2 > *C*5 > *C*4 > *C*1 > *C*3 (Fe). Moreover, the vertical profiles of trace elements (without Cd) in the sediment cores were generally similar (Fig. 4). The computed coefficients of variation (CV) for individual elements were as follows: Cd (65%) > Zn (42%) > Mn (39%) > Cu ≈ Cr (38%) > Pb ≈ Ni (35%) > Fe (26%). The greatest variation in the content of elements was found in core *C*1 (CV = 40 Fe—58% Cu) and *C*3 (CV = 29 Fe—98% Cd), and the lowest in core *C*5 (CV = 7 Fe—28% Cd). The values of coefficients of variation (CV) for trace metals in cores *C*2, *C*4, and *C*5 suggested low variation in their content. The vertical profiles in dam–reservoir sediments may be particularly valuable pollution archives (Ciszewski and Gryga 2016). Generally, trace elements display a vertical distribution pattern: Their concentration is higher in the top layers, and they gradually decrease toward deeper layers of the profile. This pattern is connected with the global process of increasing anthropogenic pressure since nineteenth century (Belzile et al. 2004). In the studies of Li and Li (2017) found that vertical distributions of Cd, Cr, Cu, Mn, Pb, and Zn in cores increased from the bottom to top layer. Harikumar and Nasir (2010) reported also higher concentration of trace metals in surface layers of sediments than in deeper ones due to development of industries and other man-made activities. In general, Gao et al. (2018) indicated the total content of trace elements decreased with depth in sediments cores, and however, in one core, metal contents remained relatively constant. On the other hand, the results of Hamzeh et al. (2013) showed that vertical distribution of metal pollution in the past is much higher than in the surface sediment. The above relationships were not identified also in this study. Figure 4 shows the trend of increasing content of trace elements (without Cd) and decreasing of their variability in bottom cores. We observed a decrease in the content and variability of Cd in bottom cores. The highest content of Cr, Ni, Cu, Mn, Fe, Zn, and Pb at depths of 4–4.5 m and 4.5–5 m, and Cd at the depths of 0–0.5 m was observed in the vertical profile (Fig. 4).

**Table 3:** Trace elements content in the sediment cores

Parameter	*C*1	*C*2	*C*3	*C*4	*C*5
	mg kg^−1^ dm				
Zn	64.1 ± 29.8a	81.6 ± 31.9a	71.7 ± 38.5a	70.4 ± 29.1a	69.8 ± 12.2a
Cu	14.6 ± 8.50a	20.6 ± 7.80c	16.3 ± 7.30ab	15.9 ± 3.66ab	19.6 ± 2.75bc
Ni	34.4 ± 17.4ab	43.2 ± 14.6b	36.8 ± 16.3ab	33.7 ± 7.58a	38.6 ± 4.28ab
Cr	26.6 ± 15.6a	30.3 ± 10.1a	26.7 ± 11.9a	23.5 ± 5.44a	26.4 ± 2.73a
Pb	12.3 ± 5.10a	15.53 ± 5.03b	12.1 ± 5.40a	11.6 ± 3.42a	12.9 ± 1.90ab
Cd	0.46 ± 0.22b	0.37 ± 0.13ab	0.32 ± 0.28ab	0.26 ± 0.13a	0.25 ± 0.07a
	g kg^−1^ dm				
Mn	0.51 ± 0.28a	0.69 ± 0.26b	0.52 ± 0.22a	0.48 ± 0.93a	0.56 ± 0.61a
Fe	13.8 ± 5.45ab	16.9 ± 3.99b	12.9 ± 3.72a	13.4 ± 2.43a	16.2 ± 1.16b

Date expression: mean ± standard deviation, a, b, c indicted significant differences between cores (*p* < 0.05)

According to Bojakowska’s geochemical quality classes of bottom sediments (2001), all of the sediment cores were classified into class II, i.e., moderately polluted sediments, due to the elevated nickel content (Bojakowska 2001). SQGs are an important tool for the assessment of contamination in bottom sediments. Those guidelines are applied for assessment of the potential ecological risk by trace elements, and they can have a high predictive ability and are a vital tool for identifying areas with potentially adverse biological effects (Hübner et al. 2009; Baran and Tarnawski 2015; Gao et al. 2018). TEC value was exceeded in most the analyzed samples only in the case of nickel. However, Ni concentration was between TEC and PEC values. The sediment samples for which metal concentrations were revealed between TEC and PEC values are potentially non-toxic, but the frequency of their toxicity occurrence is higher than for TEC value, yet lowers than for PEC (Macdonald et al. 2000; Baran and Tarnawski 2013). For other metals, TEC value did not exceed in either of the cores (Fig. 4). The high portion of nickel is generally bound to stable geochemical fractions, which suggests its limited potential bioavailability (Szarek-Gwiazda et al. 2011). Moreover, Tarnawski et al. (2017) found that bottom sediments from the inlet zone of the Rożnów reservoir were characterized by high sorption capacity, which is essential for limiting migration and bioavailability of pollutants.

**Fig. 5:**
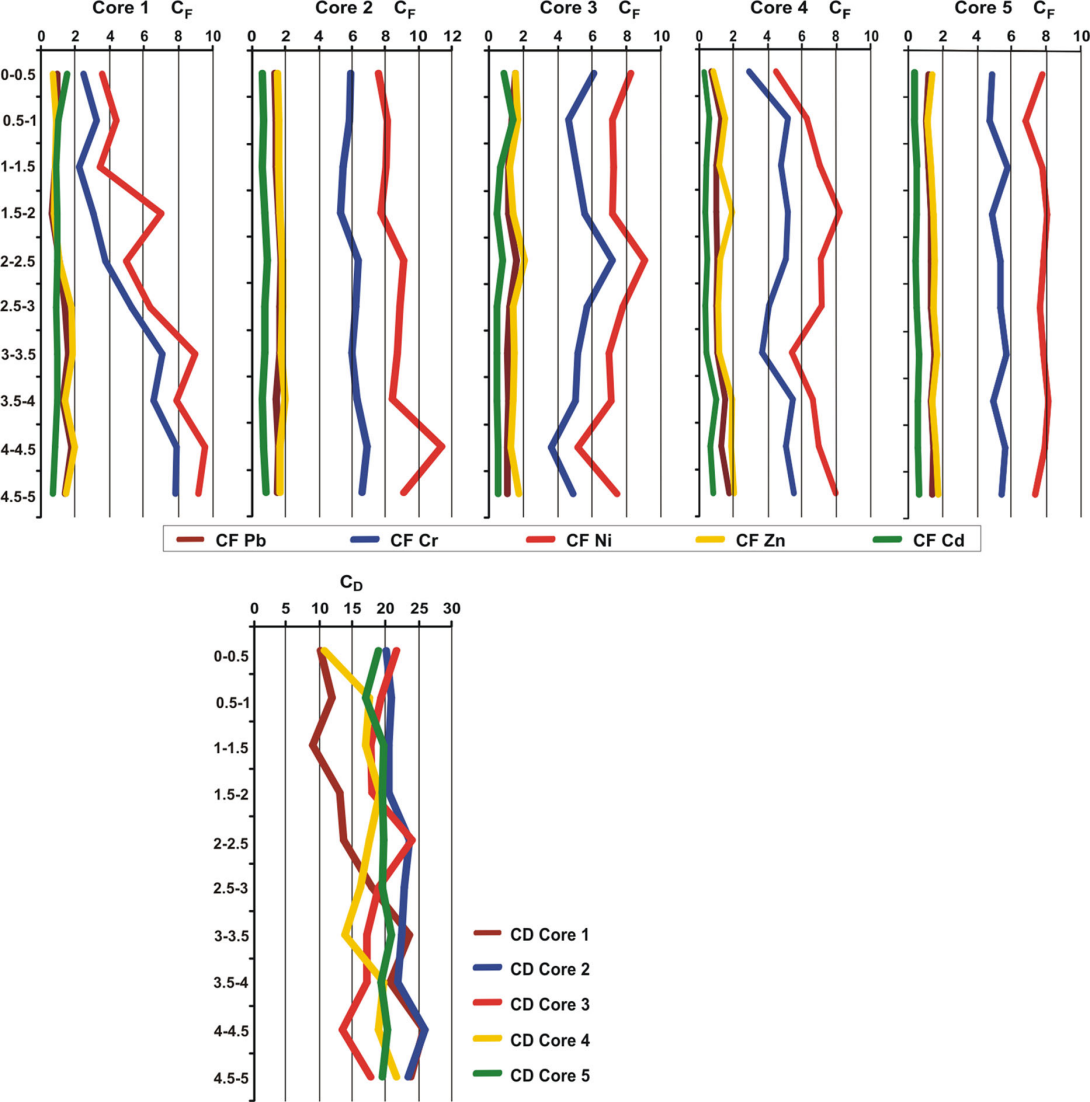
Contamination factor (CF) and contamination degree (CD) of trace elements

The important reason for using geochemical parameters is to determine the difference between trace elements of anthropogenic origin and those from natural sources (Seshan et al. 2010; Harikumar and Nasir 2010; Birch 2017). The CF values for trace elements decreased in following order: Ni > Cr > Cu > Zn > Pb > Cd (Fig. 5). The highest mean value of CF was found in core *C*2 (Cr, Ni, Cu, Zn, Pb), and Cd—in core *C*1. In the vertical profile, the highest CF values of Cr, Ni, Cu, Zn, and Pb were found at the depths of 4–4.5, 4.5–5 m, and for Cd—at the depths of 0–0.5 m, 0.5–1 m. The CF for Cu reached mean values indicating that the sediments had moderate contamination of copper. The mean value of CF for Zn and Pb was between 1 ≤ CF < 3 and for Cd CF < 1, which is indicative of moderate and low sediment pollution with these elements (Fig. 5). CF results showed that Ni, Cr, and Cd (sediment core *C*1) are the main metals that can cause relatively high contamination in the sediments. However, the mean value of CF for Ni was higher than 6, and for Cr, it was 3 ≤ CF < 6, suggesting that these trace elements had anthropogenic sources. According to the results of total degree of contamination (CD), sediment cores fall under the category of considerable contamination of metals. Values of contamination degree were between 16 ≤ *C*_D_ < 32 except for core *C*1 (0–2.5 m), core *C*3 (4–4.5 m), and core *C*4 (0.5–1 m; 3–3.5 m) which showed moderate contamination with trace elements (Fig. 5). On the one hand, nickel is commonly used in industry, and it can be found at high concentration in freshwater areas surrounding developed urban areas, and therefore, high content of Ni in sediment is a good indicator of recent anthropogenic pollution (de Castro-Català et al. 2016). Moreover, nickel and its compounds are included in the list of dangerous priority substances in the field of water quality, which should be totally eliminated from the water environment because of highly toxic properties, susceptibility to bioaccumulation, and durability (Directive of the European Parliament and of the Council 2013/39/EU of August 12, 2013). On the other hand, Szarek-Gwiazda et al. (2011) found that elevated content of Ni (15.6–83.1 mg kg^−1^ d.m.) in bottom sediments of sub-mountain reservoirs located in the Carpathian Flysch was mainly affected by high background content of Ni. The increased level of Cr in the bottom sediments of the backwater area of the Rożnów reservoir may be the result of a large number of tanning plants operating in the Dunajec catchment area with permits for tannery sewage disposal and an unknown number of plants not included in the register (Pawlikowski et al. 2006). Chromium compounds (Cr^3+^) introduced into the river are accumulated in bottom sediments. However, high content of chromium in sediment can be dangerous for benthic organisms and the higher level of the water food chain (Augustynowicz et al. 2013; Sanyal et al. 2017). Tokatli (2017) also reported that chromium and nickel content in biotic and abiotic components were the most risky elements in the aquatic environment of Emet Stream Basin in Turkey.

The highest AF value (2.2) was observed for Cd in core *C*1. Moreover, AF values above 1 indicate an anthropogenic source of trace metals we also found in core *C*3 (without Zn) and core *C*5 (Ni, Mn, Cu) (Table 4). The lowest AF values of Cr, Ni, Cu, Mn, and Fe were found in *C*1, and for Cd, Zn, Pb—in core *C*4. However, some authors have found that trace metals enrichment in the surface layer of sediments is not necessarily of anthropogenic origin (Obhodas et al. 2006; Kljakovič-Gašpič et al. 2008). This situation can be explained by redox processes which control the exchange of metals between sediments and the water column. It is commonly known that pH controls the solubility of different trace elements within water column. Trace elements can be mobilized from sediments at low pH through oxidation of sulfide phases and oxidation of organic matter (Förstner and Salomons 2010; Baran and Tarnawski 2015).

**Table 4:** An anthropogenic factor AF for metals in cores

Core	Anthropogenic factor (AF)							
	Mn	Fe	Ni	Cu	Zn	Cd	Pb	Cr
*C*1	0.4	0.5	0.4	0.4	0.5	2.2	0.7	0.3
*C*2	1.0	0.9	0.8	0.9	0.9	0.7	0.9	0.9
*C*3	1.3	1.3	1.1	1.7	0.9	1.7	1.4	1.2
*C*4	0.6	0.6	0.6	0.4	0.4	0.4	0.4	0.5
*C*5	1.0	0.9	1.1	1.0	0.8	0.8	0.9	0.9

### Ecotoxicity of bottom sediment cores

**Fig. 6:**
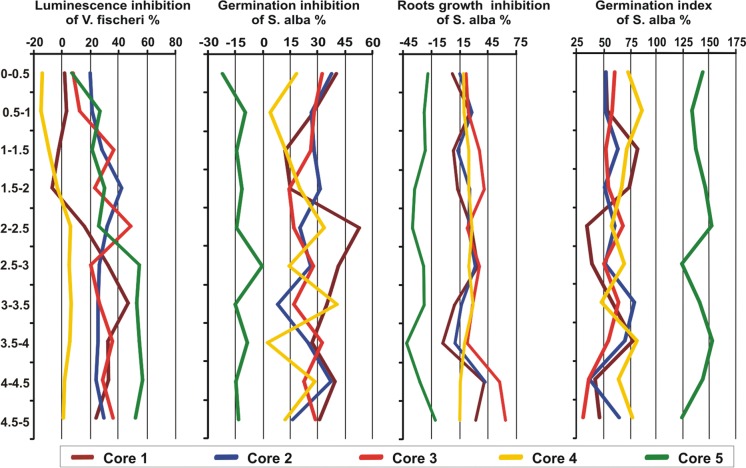
Vertical profile of ecotoxicity in the sediment cores

**Fig. 7:**
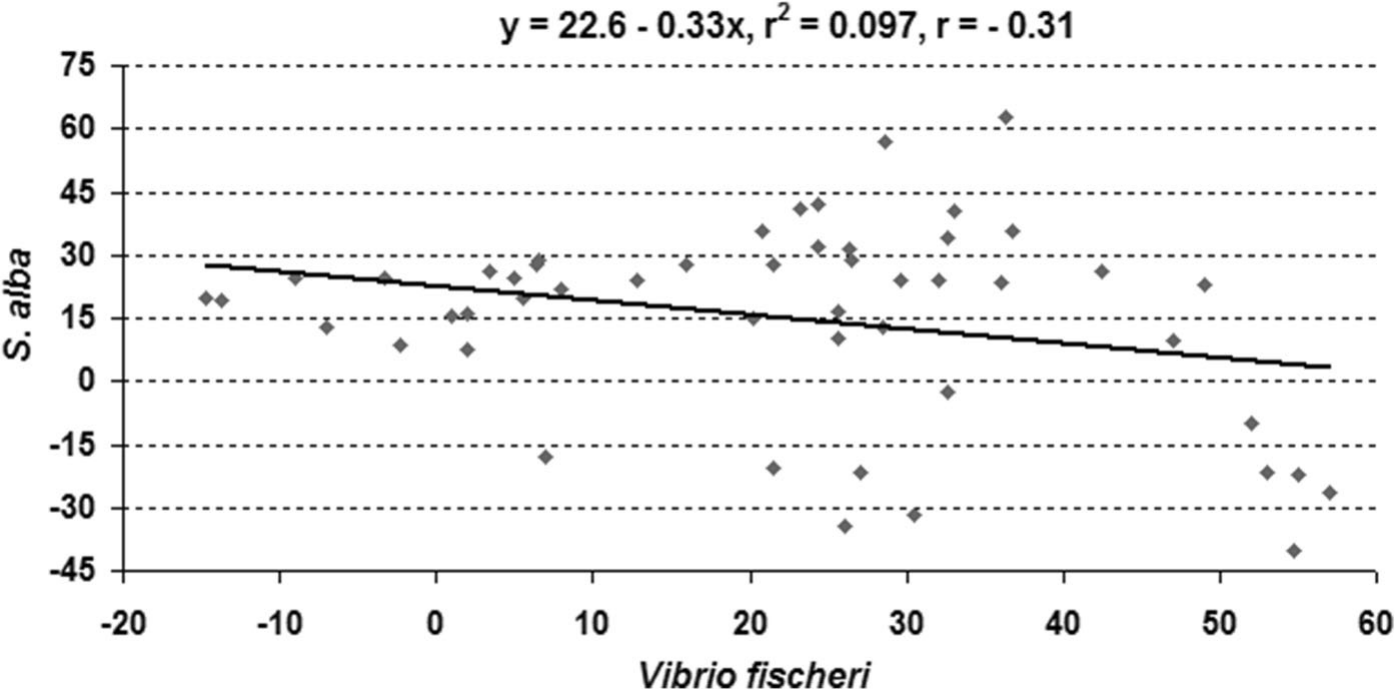
Data pairs correlations of the luminescence inhibition (%) of *V. fischeri* and roots growth inhibition (%) of *S. alba* in the sediment cores

The results point to significant differences in the organism responses of sediments collected from five cores (Table 5). Germination inhibition in *S. alba* was from − 22 to 53%, whereas root growth inhibition ranged from − 23 to 63%. Depending on the studied cores, *V. fischeri* luminescence inhibition was from − 28 to 57%. Germination index values of *S. alba* in the sediment cores ranged from 30 to 152% (Fig. 6). Growth stimulation of *S. alba* was found in core *C*5. However, other samples of the core showed growth inhibition of *S. alba* (GI > 90%) (Table 5). All cores were characterized by high variation in toxicity for the test organisms (CV = 40–162%). The highest variability was observed in vertical cores *C*4 and *C*1, and for *V. fischeri* response. We found significantly the highest number of non-toxic responses for *V. fischeri* in core *C*4, while for *S. alba*—in core *C*5. The highest toxicity of the bottom sediments in the Microtox test was observed in core *C*5 at the depth of 4–4.5 m and 4.5–5 m. Sediments collected in core *C*1 at the depth of 2–2.5 m (germination) and core *C*3 at the depth of 4.5–5 m (root growth) showed the highest toxicity in the Phytotoxkit test. Generally, higher toxic responses were recorded in the Microtox test than in the Phytotoxkit test. Ten percent of the sediment samples were toxic for *V. fischeri*, whereas two (germination inhibition), and four percent (root growth inhibition) of the samples were toxic for *S. alba*. However, most of the examined bottom sediment samples (52% for luminescence and germination inhibition, 50% for root growth inhibition) were classified as slightly toxic. Moreover, 46% of the samples (each parameter) for *S. alba* and 38% of the samples for *V. fischeri* were classified as non-toxic. In this study, negative correlations (*r* = − 0.31, p < 0.05) were found between the inhibition of luminescence and the phytotoxicity assays, so it would seem that correlation analysis showed a different sensitivity to chemicals in the sediment cores (Fig. 7). Earlier studies also found differences between the inhibition of luminescence and plant responses (Devesa-Ray et al. 2008; Garcia-Lorenzo et al. 2009; Baran and Tarnawski 2013).

**Table 5:** Toxicity of core sediments

Parameter	PE %				
	*C*1	*C*2	*C*3	*C*4	*C*5
LI^1^	18 ± 20b	28 ± 20b	27 ± 25b	− 6 ± 10a	37 ± 30b
IG	33 ± 17b	26 ± 16ab	25 ± 23ab	17 ± 14a	− 12 ± 7a
RGI	20 ± 18b	23 ± 16b	34 ± 23b	20 ± 11b	− 25 ± 10a
GI	55 ± 25a	57 ± 20a	54 ± 34a	71 ± 47a	141 ± 20b

Date expression: mean ± standard deviation, a, b, c indicted significant differences between cores (*p* < 0.05)

^*1*^*LI* luminescence inhibition, *IG* germination inhibition, *IGR* roots growth inhibition, *GI* germination index

### Correlation and PCA analysis

#### Correlation between chemical parameters

Content and distributions of nutrients and trace metals in the sediment vertical profile are controlled by numerous factors such as particle size, organic matter content, pH and redox potential, carrier substances (e.g., hydroxides, carbonates, sulfides) (Hu et al. 2013; Gao et al. 2018). In this study, we found that the content of trace elements was positively correlated with TOC (expect Cd) (Table 6). However, significantly positive correlation between the TOC content was observed only for Zn, Cu, and Fe. Positive relationships between TOC and trace element contents have often been associated with the adsorption and complexation of elements by organic matter of both autochthonous and terrestrial origin (Kljakovič-Gašpič et al. 2008; Baran and Tarnawski 2015). Moreover, the highly positive associations of elements with TOC content indicate that the distribution of metals is under the influence of TOC rather than an anthropogenic source (Hu et al. 2013). However, in heavily polluted bottom sediments, the positive correlations of trace elements with organic matter content implied their anthropogenic source (Baran and Tarnawski 2015). The relatively low content of TOC and low values of correlation coefficients suggest that TOC is not the main factor affecting the content of trace elements in the studied sediment cores. In this study, we found that trace elements were negatively correlated with pH, which means that high pH of sediments may lead to low mobility of metals. Bottom sediment reaction may significantly affect trace elements mobility: the lower the pH value, the higher the solubility of metals. Du Laing et al. (2009) and Gao et al. (2018) found that low pH decrease the negative surface charges of sediments colloids, causing the solubility and mobility of trace elements co-precipitated with carbonates and sulfides. We believe that the solubility of trace elements can be limited due to the neutral and alkaline reaction of sediments form the Rożnów reservoir. Moreover, the geochemistry of iron and manganese affects the behavior and fate of metals in water environment (Harikumar and Nasir 2010; Wang et al. 2012). Metals are efficiently adsorbed and co-precipitated with iron and manganese oxides/hydroxides (Al-Mur et al. 2017). The study of Gasparatos (2013) and Gao et al. (2018) recognized that Fe and Mn oxides/hydroxides play an important role in controlling the behavior of trace element pollution in sediments, a decrease in redox in the sediments would reductive dissolution of Fe and Mn oxides, which provide to release of metals. In our studies, we found a positive correlation of different trace elements with iron and manganese, which might indicate a redox condition in the studied sediments. The high significant positive correlation between trace elements Fe and Mn content suggested that oxidized and reduced condition may be a key factor affecting distribution and fate of metals in the analyzed cores. Many authors have suggested that positive correlations between individual pairs of trace elements indicate that they have a similar source and identical behavior in water environment (Seshan et al. 2010; Hu et al. 2013; Al-Mur et al. 2017). Moreover, our results indicate that trace elements were accumulated simultaneously in the bottom sediments and their concentrations were characterized by significantly low variability. The above is validated by significant positive correlation between pairs of metals in the sediment cores (except Cd) (Table 6) and confirm their identical origin, partly natural and anthropogenic in the Rożnów reservoir.

**Table 6:** Correlation matrices for the chemical parameters and ecotoxicity in the sediment cores

	Cr	Ni	Cu	Zn	Cd	Pb	Fe	Mn	Na	Mg	K	Ca	P	N	S
Ni	0.83*														
Cu	**0.75**	**0.82**													
Zn	**0.66**	**0.62**	**0.68**												
Cd	0.23	0.12	0.22	0.23											
Pb	**0.82**	**0.75**	**0.91**	**0.80**	**0.39**										
Fe	**0.79**	**0.83**	**0.91**	**0.66**	0.16	**0.86**									
Mn	**0.86**	**0.87**	**0.89**	**0.63**	0.22	**0.87**	**0.89**								
Na	**0.76**	**0.77**	**0.82**	**0.64**	0.27	**0.83**	**0.91**	**0.87**							
Mg	**0.48**	**0.60**	**0.74**	**0.47**	− 0.09	**0.61**	**0.81**	**0.64**	**0.80**						
K	**0.60**	**0.69**	**0.84**	**0.63**	0.06	**0.77**	**0.89**	**0.74**	**0.89**	**0.93**					
Ca	0.14	0.24	**0.50**	**0.33**	− 0.12	**0.39**	**0.48**	**0.38**	**0.54**	**0.82**	**0.72**				
P	**0.33**	0.11	0.09	0.18	**0.75**	**0.31**	0.01	0.23	0.17	**− 0.30**	− 0.14	**− 0.29**			
N	− 0.01	0.12	**0.34**	**0.33**	**− 0.33**	0.26	**0.29**	0.11	0.18	**0.47**	**0.46**	**0.48**	**− 0.53**		
S	**0.36**	**0.38**	**0.63**	**0.38**	0.04	**0.58**	**0.62**	**0.50**	**0.63**	**0.75**	**0.73**	**0.62**	− 0.17	**0.42**	
pH	− 0.21	− 0.22	**− 0.31**	**− 0.37**	− 0.06	**− 0.33**	**− 0.42**	− 0.28	**− 0.46**	**− 0.52**	**− 0.57**	**− 0.43**	0.15	**− 0.40**	− 0.26
TOC	0.06	0.10	**0.35**	**0.35**	**− 0.31**	0.25	**0.30**	0.11	**0.25**	**0.65**	**0.55**	**0.78**	**− 0.47**	**0.65**	**0.47**
IG^a^	0.06	− 0.05	− 0.17	0.00	**0.33**	− 0.01	− 0.19	− 0.05	− 0.12	**− 0.46**	**− 0.38**	**− 0.38**	**0.58**	**− 0.32**	**− 0.48**
RGI	0.05	− 0.03	− 0.17	0.02	**0.22**	− 0.04	− 0.25	− 0.04	− 0.27	**− 0.52**	**− 0.40**	**− 0.52**	**0.29**	− 0.10	**− 0.48**
IL	**0.47**	**0.46**	**0.44**	**0.32**	0.01	**0.49**	**0.51**	**0.49**	**0.50**	**0.44**	**0.47**	0.22	− 0.02	0.24	**0.58**

*The bold values indicate significant at the 0.05 level

^a^*IG* germination inhibition, *RGI* roots growth inhibition, *IL* luminescence inhibition

The content of nutrients (N, S, Ca, Mg, and K) were also significantly positively correlated with TOC content and significantly negatively correlated with pH (Table 6). A high significant correlation between TOC and N, Mg, Ca, and S indicates that the main origin of these nutrients is the mineralization of organic matter (Jung 2017). In the sediment cores, a high positive correlation was evidenced between pairs of nutrients (except P). Moreover, we found significant positive correlation between nutrients and trace elements, which point to common pollution or similar sources and migration process. For Cd and P, a different relationship has been shown. Both elements were significantly negatively correlated with TOC. Moreover, relationships among the cadmium and phosphorus was significantly positively correlated (*r* = 0.75), indicating that these elements had the same natural and anthropogenic sources. For both elements, their highest content was found in sediment core *C*1 (Figs. 3, 4).

#### Correlation between chemical properties and ecotoxicity of sediment cores

The correlations between the trace elements and nutrients content in the cores and the toxicity results are shown in Table 6. Generally, a positive correlation indicates a relation between elements content and toxicity to organisms, whereas negative values of the correlation coefficients indicate that the content of an element did not affect the sediment toxicity. The correlation analysis recorded a significant positive relation between inhibition of luminescence of *V. fischeri* and the content of trace elements (Cr, Ni, Zn, Pb, Mn, and Fe) and nutrients (K, Mg, Na, S, and N). However, values of the correlation coefficients were low and middle, suggesting a weak relationship. For *S. alba,* a significant negative correlation between the content of nutrients (except P) and inhibition of germination and root growth was observed (Table 6). For trace elements and *S. alba*, the correlations, being no statistically significant (positive Cr, Zn and negative Ni, Cu, Fe, Mn), are characterized by a very low correlation coefficient. However, the inhibition of germination and root growth of *S. alba* in the sediment cores correlated significantly positively with Cd and P (Table 6). This observation is in concordance with our previous studies associated with analysis ecotoxicity of bottom sediments form Rzeszów reservoir (Tarnawski and Baran 2018). Płaza et al. (2010) and Baran et al. (2016) also observed a significant positive correlation between the content of trace elements in soils and sediments and the inhibition of *V. fischeri.* Research carried out by Baran and Tarnawski (2013) found a positive correlation between the content of trace elements in bottom sediments and root growth inhibition of *L. sativum* as well as luminescence inhibition of *V. fischeri*. In the studies of Fafandel et al. (2015), no significant correlation of nutrients and toxicity for *V. fischeri* was observed. However, the authors found that the highest toxicity of sediments for bacteria was associated with high nitrogen input form agriculture activities and uncontrolled waste disposal (Fafandel et al. 2015). In the studies of Janke et al. (2011), it was observed that the main chemical substance that probably caused the elevated toxicity to *Ceriodaphnia silvestrii and Chironomus xanthus in sediment pore water and bottom sentiments* was nitrate. Gao et al. (2018) found that not total concentrations of trace elements in sediments were responsible for sediment toxicity, but acid-soluble forms contributed significant toxicity for *V. fischeri* in the sediments. In the study, not all sediment samples gave a toxic response of tested organisms. The organic matter, total nitrogen, and other nutrients are essential for growth of producers in the aquatic ecosystem (Kundrat et al. 2017; Jung 2017). In our studies, it was demonstrated that the some sample of sediments had stimulant effects of plants growth. Kundrat et al. (2017) found that the high mineral level of the bottom sediments had stimulant effect on seed germination and roots growth of *S. alba.* Czerniawska-Kusza et al. (2006) also demonstrated that higher amount of organic matter and nutrients in bottom sediments stimulated root growth of *S. alba* and *L. sativum.* The phenomenon that chemicals can be stimulatory (at low concentrations) to organisms has been named “hormesis” (Belz and Cedergreen 2010). Samples showing hormesis are currently non-toxic. In our previous studies, we found hormesis in bottom sediments from Zesławice reservoir (Baran and Tarnawski 2013) that were moderately contaminated with heavy metals. The problem associated with growth stimulation, especially of test plants, may occur in the case of sediments rich in biogenic and fertilizing substances (carbon, nitrogen, phosphorus, sulfur, calcium, magnesium, and potassium), which may act as a stimulant for plant growth even with significant content of toxic substances, or in the case of an intensive eutrophication process where their toxicity may be reduced by biosorption (Baran et al. 2016). Other authors have observed hormesis after exposure to metals with luminescent bacteria and to herbicides with plants (Belz and Cedergreen 2010).

#### Multivariate analyses

**Fig. 8:**
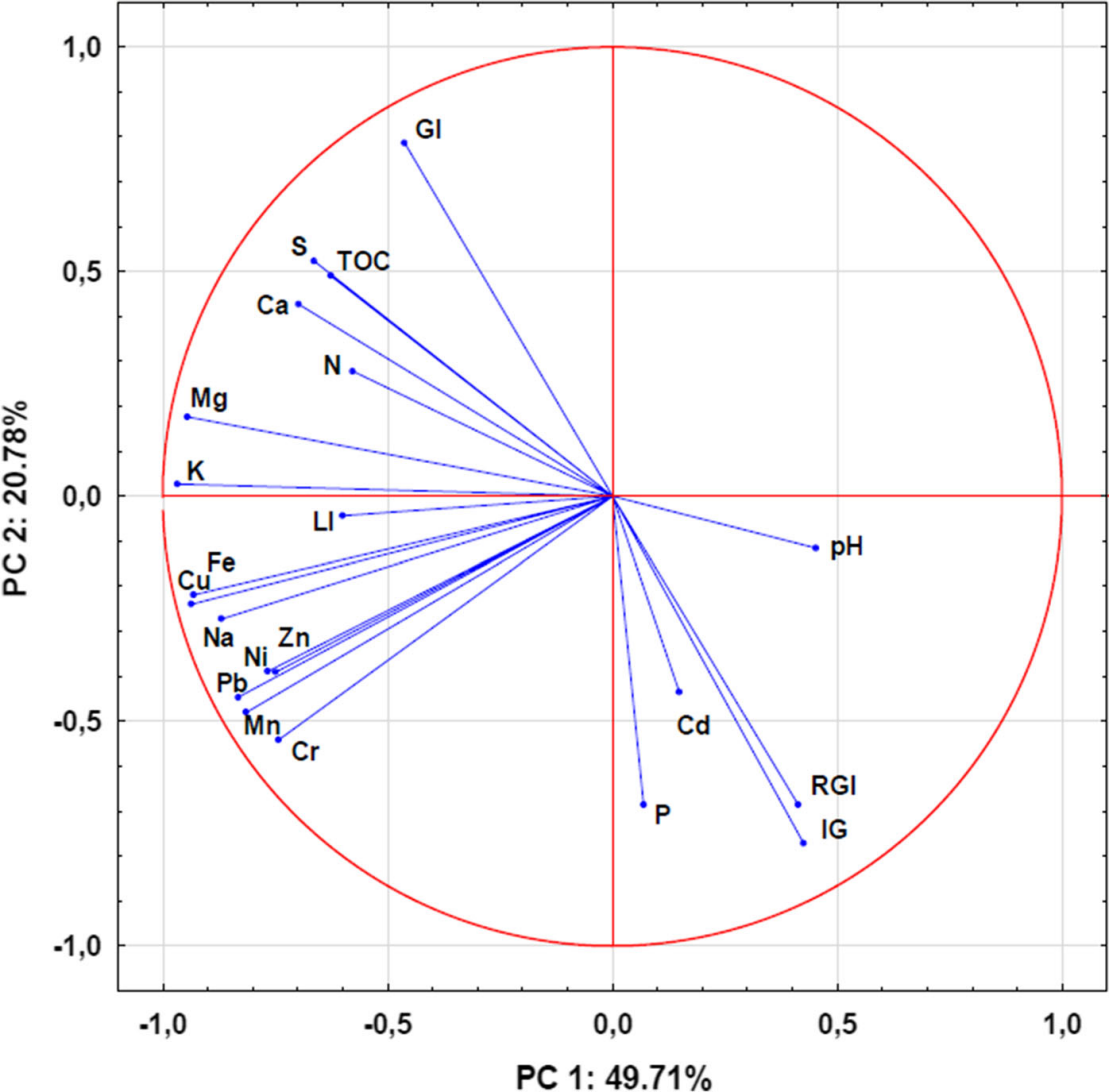
Principle component analysis of trace elements, nutrients, and toxicity of sediment cores. *LI* luminescence inhibition, *IG* germination inhibition, *RGI* roots growth inhibition, *GI* germination index

Principal component analysis (PCA) confirmed the above observations and allowed to find other interesting relationships between the investigated variables (Fig. 8). The two PCs factor described 70.49% of the variances. The first factor PC1 accounted 49.71% of the variances and was strong negatively correlated with the trace elements, K, Mg, and Na, less with N, S, TOC, and inhibition of luminescence of *V. fischeri* (LI). The second factor PC2 explained 20.78% of variance with high positive loading on germination index (GI), less on TOC, N, S, and Ca, and negatively correlated to inhibition of germination and roots growth of *S. alba* (IG, RGI), P, Cd, and Cr (Fig. 8). These results indicated that PC1 had similar geochemical behaviors and mixed sources and are possible controlled by hydrous iron and manganese oxides. Hydrous iron and manganese oxides are known to important solid phases for adsorption and transporting of trace elements in aquatic bottom sediments (Förstner and Salomons 2010). The second PC2 had different behaviors and also natural and anthropogenic sources. Cadmium and phosphorus were associated with toxicity for *S. alba,* whereas TOC, N, S, and Ca for stimulation of growth this plants. In general, the results of long-term studies show that the Rożnów reservoir has eutrophic character (Szarek-Gwiazda 2014). Moreover, inflow of nutrients into the reservoir is high due to soil erosion, insufficient municipal wastewater treatment, and village wastewaters (lack of sewage system in villages around the reservoir). However, the bottom sediments form the backwater zone of the Rożnów reservoir had relatively low content of TOC and nutrients (N, P, and S) and mineral character. The previous study showed dominance of silt and clay fraction in bottom sediments of the backwater zone of the Rożnów Reservoir (Tarnawski et al. 2017). Other authors have also found that bottom sediments from the Rożnów reservoir are rich in clay and silt fraction and contain small amount of organic carbon, nitrogen, and phosphorus (14–21 g/kg TOC; 1.0–3.1 g/kg N; 0.07–1.57 g/kg P) (Szarek-Gwiazda and Mazurkiewicz-Boroń 2010; Szarek-Gwiazda 2014). The lack of high content of organic carbon and nutrients in cores is caused by the rheolimnetic character of the reservoir and the high content of suspended matter transported with the Dunajec river. Silting process is one of the main factors limiting the proper use of water reservoirs and also affecting the quality of their waters. It is responsible for the inflow of fine (mineral or organic) fractions of both natural and anthropogenic origin (Tarnawski et al. 2017). The Rożnów reservoir, especially its backwater area, is the fastest silting dam reservoir in Poland (Szarek-Gwiazda and Mazurkiewicz-Boroń 2010; Szarek-Gwiazda 2014; Tarnawski et al. 2017). The research conducted by Tarnawski et al. (2015) shows that, after 50 years of operation, there is over 16 million m^3^ of sediment in the inlet area of the reservoir. Analyses of the possibility of restoring the storage capacity of the reservoir show the necessity for carrying out works for nearly 40 years generating huge costs. The inlet zone of the reservoir is particularly intensely shallowed through the silts of the Dunajec river. The main factors affecting the silting process of the Rożnów reservoir include: the geological structure of the catchment area, frequent floods, surface water from agricultural fields and municipal sewage flowing directly into the reservoir. In addition, the Dunajec River, on which the reservoir was created, is a receiver of contaminants from smaller rivers and streams, i.e., its tributaries, is the place where both industrial and municipal sewage are discharged. In the geological structure of the Rożnów reservoir catchment area, the largest area is occupied by the Carpathian Flysch Belt made of sandstones, conglomerates, and Paleogene and Upper Cretaceous shales—material easily eroded from the ground, representing the main source of silting of the reservoir. The results also confirmed that sediments of reservoirs created on Carpathian Flysch are characterized by high content of calcium in bottom sediments. High content of calcium in the sediment is associated with high concentrations of HCO_3_^−^ and Ca^2+^ ions in the water of the Dunajec river supplying the studied reservoir (Szarek-Gwiazda 2014). Two of the main threats related to intensive silting of the reservoir are the eutrophication process and the pollution of the bottom sediments with trace elements (Szarek-Gwiazda 2014). The silting process contributes to the inflow of fine fractions of both natural and anthropogenic origin is important source of trace elements and nutrients in the sediment core. Fine fractions have high sorption capacities for trace elements (Burton et al. 2004; Jung 2017). Moreover, the clay and silty content is the dominant fraction in the bottom sediments of the Rożnów reservoir (Tarnawski et al. 2017).

## Conclusion

The obtained data results found generally significant differences in the content of nutrients, trace elements, and ecotoxicity between five sediment cores. However, there was no high variability of the above parameters in the vertical distribution. This means that the intensely suspended matter transported by the Dunajec river is and, at various times, has been homogeneous. Moreover, significant correlations between nutrients and trace elements point to the same sources of the above-mentioned substances and similar levels of contamination in the sediment cores. The intensive silting process that contributes to the inflow of fine fractions is a significant source of trace elements and nutrients in the sediment core. However, the PCA results found that cadmium and phosphorus in the sediment cores originate from different sources than other elements and can be associated mainly with anthropogenic sources. According to the degree of contamination factor, sediment cores fall under the category of considerable contamination of metals. In this study, nickel, chromium, and cadmium (sediment core *C*1) were found to be as the significant substances of polluting in the sediment cores. The main sources of Ni and Cr in sediments are wastewater, leather tanning industries (located in the Dunajec catchment), and natural processes. However, according to the sediment quality guidelines (SQGs), it was determined that neither nickel nor chromium or other trace elements caused adverse biological effects. Toxicity assessment found that most of the bottom sediment samples were classified as non-toxic or slightly toxic, but the two test organisms showed a different sensitivity. Higher toxic responses were recorded in the Microtox with *V. fischeri* than in the Phytotoxkit test with *S. alba*. However, only 10% of the sediment samples were toxic for *V. fischeri*, and 6% of the samples were toxic for *S. alba*. The correlation analysis and PCA results showed that trace elements and nutrients content were associated with toxicity of sediments toward test organisms. Cadmium and phosphorus were associated with toxicity for *S. alba,* whereas TOC, N, S, and Ca for stimulation of growth this plants. Trace elements and nutrients (S, K, Mg, and Na) were positively correlated with inhibition of luminescence of *V. fischeri*. The results of this study support the idea that bottom sediments are complex matrix and assessment of their toxicity derived not only from measured content of trace elements and nutrients, but also from the their interaction and multiple sources or other factors, not measured in this study. Moreover, we found that sediment toxicity could be related to nutrients content in sediments of Ronżnów reservoir since risk assessment based on trace elements concentration revealed low or moderate potential risk for the benthic organisms. Therefore, the evaluation of nutrients, trace elements content, and ecotoxicity in sediments represent significant part in ecological risk assessment process, and it is important for the management of bottom sediments.
